# The Importance of Metabolism for Immune Homeostasis in Allergic Diseases

**DOI:** 10.3389/fimmu.2021.692004

**Published:** 2021-07-28

**Authors:** Juan Rodriguez-Coira, Alma Villaseñor, Elena Izquierdo, Mengting Huang, Tomás Clive Barker-Tejeda, Urszula Radzikowska, Milena Sokolowska, Domingo Barber

**Affiliations:** ^1^Departamento de Ciencias Medicas Basicas, Instituto de Medicina Molecular Aplicada (IMMA), Facultad de Medicina, Universidad San Pablo-CEU, CEU Universities, Boadilla Del Monte, Madrid, Spain; ^2^Centre for Metabolomics and Bioanalysis (CEMBIO), Department of Chemistry and Biochemistry, Facultad de Farmacia, Universidad San Pablo-CEU, CEU Universities, Boadilla Del Monte, Madrid, Spain; ^3^Swiss Institute of Allergy and Asthma Research (SIAF), University of Zurich, Davos Wolfgang, Switzerland

**Keywords:** allergy, immunometabolism, immune cells, metabolic regulation, -omics

## Abstract

There is increasing evidence that the metabolic status of T cells and macrophages is associated with severe phenotypes of chronic inflammation, including allergic inflammation. Metabolic changes in immune cells have a crucial role in their inflammatory or regulatory responses. This notion is reinforced by metabolic diseases influencing global energy metabolism, such as diabetes or obesity, which are known risk factors of severity in inflammatory conditions, due to the metabolic-associated inflammation present in these patients. Since several metabolic pathways are closely tied to T cell and macrophage differentiation, a better understanding of metabolic alterations in immune disorders could help to restore and modulate immune cell functions. This link between energy metabolism and inflammation can be studied employing animal, human or cellular models. Analytical approaches rank from classic immunological studies to integrated analysis of metabolomics, transcriptomics, and proteomics. This review summarizes the main metabolic pathways of the cells involved in the allergic reaction with a focus on T cells and macrophages and describes different models and platforms of analysis used to study the immune system and its relationship with metabolism.

## Introduction

The correct onset and regulation of immune responses in cancer and infectious diseases are affected by the activation of different intracellular metabolic pathways. When we talk about the sequence of metabolic events that takes place in different immunological cellular players, we refer in a general way to immunometabolism (1). There is an increasing interest in figuring out the changes in metabolic routes associated with the proliferation and control of immunological responses. Due to the SARS-CoV-2 pandemic, metabolic co-morbidities such as diabetes and obesity have dramatically been associated with an increased risk for severe disease, highlighting the relevance of energy metabolism for mounting correct immunological responses to infections (2–4). Another area where immunometabolism research has exploded in recent years is cancer. Metabolic reprogramming of the immune system by cancer cells (5) and new intervention strategies aiming to reactivate anergic cells or to block tumor-induced regulatory signals are subjects of intensive research (6–8).

Cells adapt their metabolic status by means of sensors detecting extracellular or intracellular signals. Determination of the mechanisms involved will provide new potential therapeutic targets to control inflammation. In fact, already today, different therapeutic approaches such as rapamycin, calcineurin inhibitors, or purine and pyrimidine synthesis inhibitors target T cell proliferation to alleviate autoimmune or inflammatory diseases (9).

Little is known about the intracellular metabolic changes in cells that lead to exacerbated allergic responses to innocuous antigens. The variety in exposome, from mere allergic sensitization with occasional exposure (as in the case of Hymenoptera venom allergy) to allergic perennial phenotypes in highly poly-sensitized patients makes allergic diseases an interesting and complex model to analyze the role of an altered energy metabolism in disease progression. Recent evidence suggests that metabolic reprogramming steering T cell proliferation plays an important role in the evolution from mild to severe allergic phenotypes (10, 11). In fact, systemic signatures pointing to Warburg metabolism have been described in severe allergy respiratory patients (12). Therefore, elucidating the mechanisms underlying the metabolic rewiring of immune cells might be a key to understand the evolution to severe allergic phenotypes and to develop new biomarker strategies that could lead to a personalized intervention, a clear unmet need in allergic patient management (13, 14). For this purpose, it is imperative to understand the values and limitations of the methodological approaches used to study immunometabolism as it ranges from more classical immunological and biochemical approaches like flow cytometry to the single-cell level of different -omics studies.

In the present review, we provide a holistic view of immunometabolism with a focus on allergic diseases. We provide a general view of metabolic routes at both systemic and cellular levels, with a focus on the main immunological players, and summarizing the main methodological approaches used so far.

## The Immune System of the Allergic Reaction

The classical allergic reaction is an immune response characterized by the leading role of allergen-specific type 2 T helper cells (Th2) and type 2 innate lymphoid cells (ILCs), which produce their characteristic cytokines – mainly interleukin (IL)-4, -5, and -13 (15, 16). These cytokines lead to an inflammatory environment with the involvement of other cell types, mainly epithelial cells of the skin and mucosa, dendritic cells (DCs), mast cells (MCs), basophils, and eosinophils (17, 18).

The most prevalent and well-known allergic responses are type-2 reactions, which are immunoglobulin E (IgE)-dependent (i.e., allergic asthma, anaphylaxis, allergic rhinitis, atopic dermatitis, and most food allergies) (17). However, other allergic phenotypes with dominance on non-type 2 inflammation (or so-called type-2 low) and no significant IgE response are also quite frequent, such as non-IgE-mediated food allergies or type-2 low asthma (19). Their mechanisms are much less studied (20).

**Figure 1:**
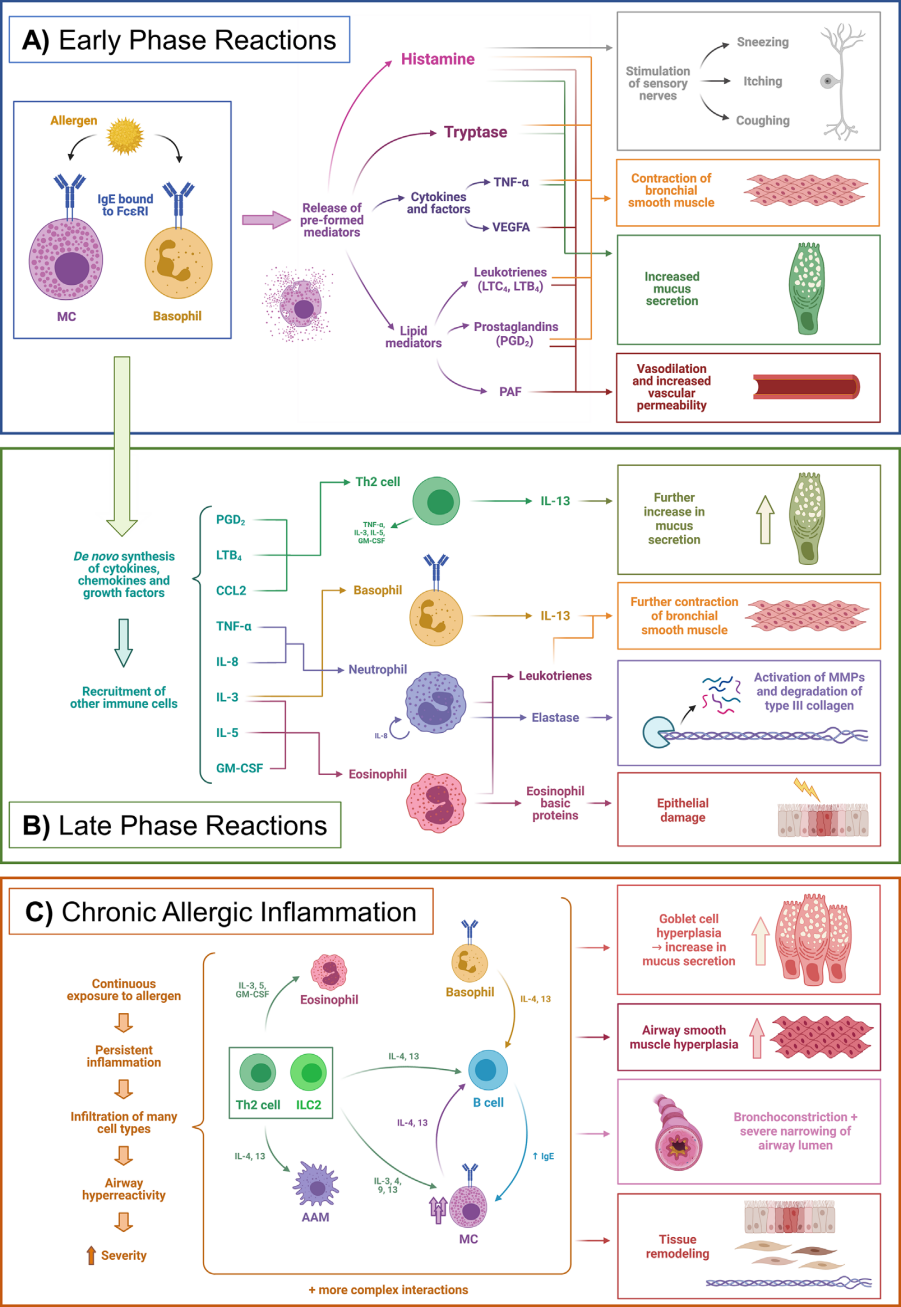
Phases of allergic inflammation. Example of a simplified allergic asthma model. **(A)** Early phase reactions, **(B)** Late phase reactions, **(C)** Chronic allergic inflammation. AAM, Alternatively activated macrophages; CCL2, Chemokine (C-C motif) ligand 2; FcεRI, High-affinity IgE receptor (Fc epsilon receptor I); GM-CSF, Granulocyte-macrophage colony-stimulating factor; IgE, Immunoglobulin E; IL, Interleukin; ILC2, Type 2 innate lymphoid cell; LTB_4_, Leukotriene B4; LTC_4_, Leukotriene C4; MC, Mast cell; MMPs, Matrix metalloproteinases; PAF, Platelet-activating factor; PGD_2_, Prostaglandin D2; Th2, Type 2 T helper cell; TNF-α, Tumor necrosis factor alpha. Adapted from Galli et al. Nature 2008;454 (7203),445-454. Created with BioRender.com.

There are three main phases in the course of allergic inflammation: early-phase reactions, late-phase reactions, and chronic allergic inflammation (**Figure 1**) (21, 22).

Early phase reactions are characterized by the liberation of mediators from MCs – and basophils to a lesser extent (23). For this, sensitization must have previously occurred, in which the allergen has been presented to T cells by antigen-presenting cells (APCs) mainly DCs and macrophages. Briefly, APCs ingest allergenic proteins, process them to peptides, and present them *via* the major histocompatibility complex class II (MHC-II) to naïve T cells in the lymph nodes. Then, this presentation triggers a type 2 polarization which ultimately results in the production of allergen-specific IgE by plasma cells, which are derived from the immunoglobulin class-switch recombination and differentiation of B cells (21, 24). The role of epithelial cells as essential components of the innate immune response and specifically in allergy has sparked increasing interest and has been reviewed recently (17). This IgE is bound to the high-affinity Fc epsilon receptor I (FcεRI) on the surface of MCs and basophils. When re-exposed to the sensitized allergen, binding to the IgE triggers the release of the aforementioned mediators, which have been pre-formed and stored in cytoplasmic granules. These are mainly histamine, serine proteases such as tryptases, other enzymes, cytokines [e.g. tumor necrosis factor-alpha (TNF-α) and vascular endothelial growth factor A (VEGFA)], and many potent lipid mediators, such as prostaglandin D_2_ (PGD_2_), leukotriene B4 (LTB_4_) and cysteinyl leukotrienes (CysLTs) (**Figure 1A**) **(** 21, 25, 26). If the mediators are released locally and in a self-limiting way, the symptoms are usually not life-threatening, whereas a rapid and massive release into the circulation may cause a severe systemic reaction called anaphylaxis (21, 27).

Apart from these pre-formed mediators, MCs also produce cytokines, chemokines, lipid mediators, and growth factors *de novo* when triggered by an allergen. This process is slower than the fast degranulation, and the consequences are evident in the late-phase reaction, occurring hours after the allergen challenge. This is a consequence of the recruitment of other immune cells by products such as TNF-α, IL-8, chemokine (C-C motif) ligand 2 (CCL2), CysLTs, and other chemokines. Cells recruited include Th2 cells, neutrophils, monocytes, eosinophils, and basophils (**Figure 1B**) (28). However, late-phase reactions do not occur in all patients and may not be clearly delimited from early-phase reactions (21).

If the exposure to the allergen persists over time, or the inflammation is not resolved adequately, the immune response evolves into chronic allergic inflammation, which is characterized by infiltration of many different types of type 2 and non-type 2 immune cells from both innate and adaptive systems. This infiltration can ultimately lead to structural changes – i.e. tissue remodeling (29) – and altered functions of the affected organs. In the case of asthma, a well-studied example of this process, the remodeling includes thickening of the airway walls, hyperplasia of goblet cells – with the subsequent increase in mucus production – epithelial injury and increased numbers of MCs, to name a few examples, leading ultimately to a state of airway hyperreactivity and a more severe phenotype (**Figure 1C**) (19, 21, 22, 29). Additionally, persistent IgE levels – at least in lifelong food allergies– seem to be sustained by allergen-specific long-lived ${\text{IgG}}_{1}^{+}$ memory B cells that upon reactivation with an allergen undergo class-switch recombination and replenish the IgE^+^ plasma cell compartment, instead of long-lived IgE^+^ plasma cells as was previously thought (24).

## Fuel for Function: Energy Metabolism in Human Cells

**Figure 2:**
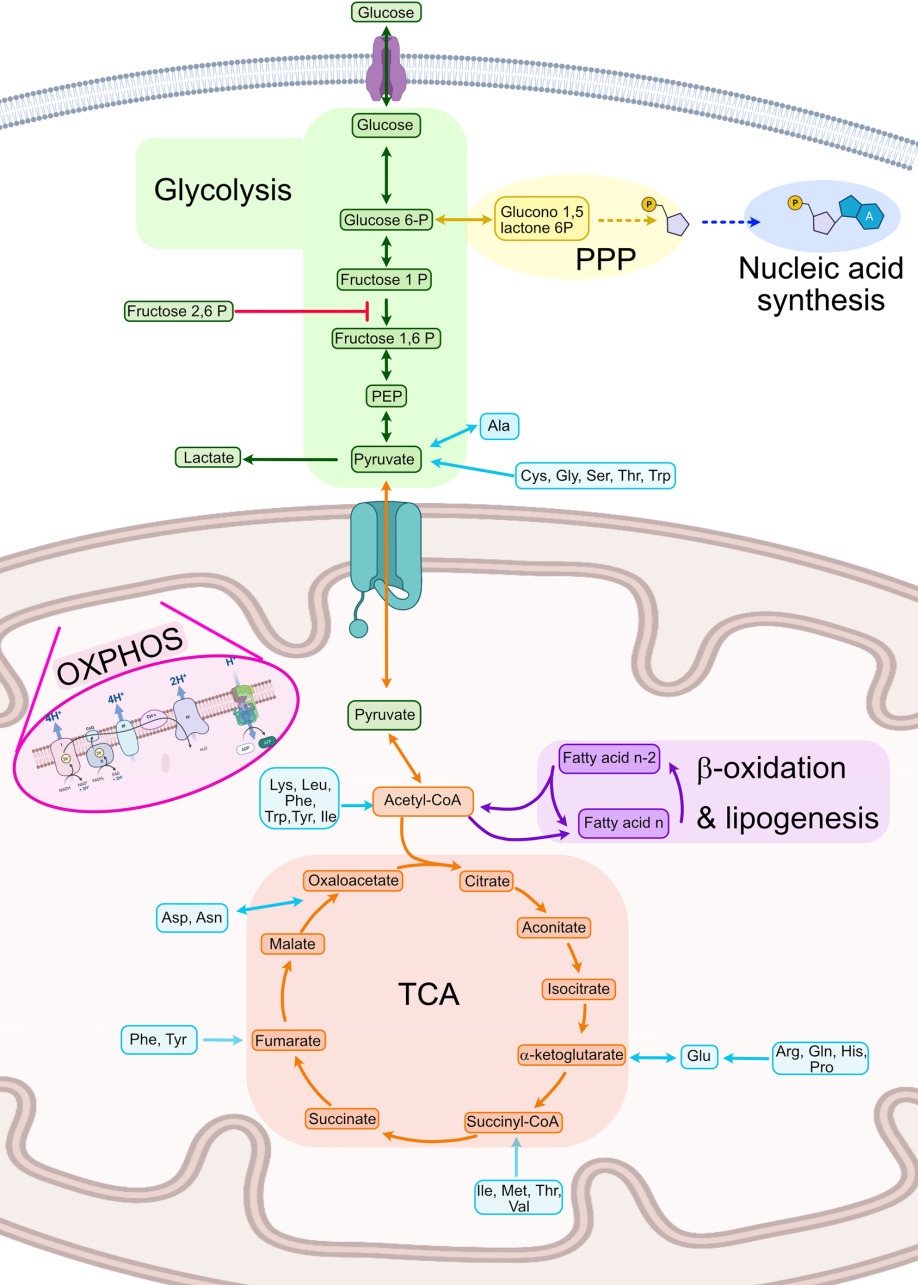
Summary of human cellular metabolism. In green steps from the glycolysis pathway, in yellow the PPP, in dark blue nucleic acid biosynthesis, in orange the TCA, in purple fatty acid oxidation and lipogenesis, in light blue amino acid metabolism. Ala, Alanine; Asn, Asparagine; Asp, Aspartate; Cys, Cysteine; Gln, Glutamine; Glu, Glutamate; Gly, Glycine; His, Histidine; Ile, Isoleucine; Leu, Leucine; Lys, Lysine; Met, Methionine; OXPHOS, Oxidative phosphorylation; PEP, Phosphoenol pyruvate; Phe, Phenylalanine; PPP, Pentose phosphate pathway; Pro, Proline; Ser, Serine; TCA, Tricarboxylic acid cycle Thr, Threonine; Trp, Tryptophan; Tyr, Tyrosine; Val, Valine. Created with BioRender.com.

Metabolism can be defined as the sum of all chemical reactions used for sustaining life. Although every type of cell presents some specific reactions, a great amount of cell metabolism is conserved between cell types. As such, this part of the review will focus on the general metabolism used for energy production and its general regulation (**Figure 2**).

One of the most important metabolic pathways for energy generation is glycolysis, which is a heavily controlled process focused on the complete breakdown of glucose molecules to obtain nicotinamide adenine dinucleotide (NADH) and energy. Hexokinases (HK) 1-3 are the first enzymes needed for initiating the pathway by phosphorylating glucose molecules and thus inhibiting glucose excretion from the cell (30, 31). Secondly, the reaction that converts fructose-P to fructose 1,6-bisphosphate (1,6-FBP), catalyzed by the phosphofructose kinase (PFK), is the main point of regulation of the glycolysis as it is an irreversible step (32–34). This enzyme is only activated in presence of fructose 2,6 bisphosphate (2,6-FBP), which is synthetized by PFK2 under high levels of intracellular adenosine monophosphate (AMP). Finally, the last step of the glycolysis pathway catalyzes the conversion of phosphoenolpyruvate (PEP) to pyruvate and ATP by pyruvate kinase (PK) which is also regulated by 2,6-FBP (35). Glycolysis is usually used in anaerobic conditions or when fast energy is required. However, in the case of activated immune cells, it is one of the most important pathways for energy production even in the presence of oxygen, a phenomenon called aerobic glycolysis or Warburg effect (36, 37). When nucleic acid synthesis is needed, G6P can be used instead to create the pentoses needed for nucleic acid synthesis using the anabolic pathway called pentose phosphate pathway (PPP) (**Figure 2**).

Once pyruvate is generated, it needs to be further processed into either lactate (anaerobiosis) or acetyl coenzyme A (acetyl-CoA) to regenerate the reductive molecules generated in glycolysis (NADH). Acetyl-CoA can enter the tricarboxylic acid cycle (TCA) also known as the Krebs cycle which takes place in the mitochondria (38). This cycle is an amphibolic pathway, meaning it can participate in both anabolic and catabolic processes depending on the needs of the cell (39). TCA is a central pathway in which different metabolic processes such as glycolysis, transamination, deamination, lipogenesis, and β- oxidation converge (39). In the TCA, acetyl-CoA is introduced into the TCA by the enzyme citrate lyase, which produces citrate. This molecule will be completely metabolized in a sequence of reactions until oxaloacetate (**Figure 2**). This cycle is mainly regulated by the concentrations of product and the availability of substrates in each step. Conversion of acetyl-CoA into citrate is usually the limiting step. TCA takes place in the mitochondria of human cells and produces ATP and NADPH. The latter is used to fuel oxidative phosphorylation (OXPHOS) also known as mitochondrial respiration. OXPHOS is based on a series of redox reactions in the inner mitochondrial membrane by the electron transport chain complex to completely reduce O_2_ to water (40). In the first step, H^+^ ions from NADH and nicotinamide adenine dinucleotide phosphate (NADPH) are pushed into the intermembrane space generating a chemical gradient and different electric potential across the inner membrane. At the same, the electrons of those H atoms get transported along a protein transport chain in the inner mitochondrial membrane. At the end of the chain, the ATPases use the H^+^ electrochemical gradient to generate ATP when the H^+^ return to the mitochondrial matrix. These protons will be then linked to O_2_ and the transported electrons to synthesize H_2_O (41) (**Figure 2**).

Acetyl-CoA can also be generated in β-oxidation also known as fatty acid (FA) oxidation (FAO), in which a series of sequential reactions convert FAs into acetyl-CoA (42). First, the FAs are activated by adding the CoA molecule by an acyl-CoA dehydrogenase. Secondly, the FAs are transported into either the mitochondria or the peroxisome by carnitines (43, 44). Thirdly, they will be sequentially dehydrogenated, hydrated, and reduced, and finally the molecule of acetyl-CoA and a FA with 2 fewer carbons (n-2 FA) are generated. In lipogenesis, the opposite procedure is carried out, in which from acetyl-CoA longer acyl chains are built up by carbon pairs until the FA reaches the desired length (**Figure 2**).

Finally, amino acids in immune cells have other functions apart from the synthesis of proteins, such as energy consumption in T cells. Amino acids can be incorporated into the TCA cycle at different points, as described in **Figure 2** (45). Specifically, ketogenic amino acids [leucine (Leu) and lysine (Lys)] enter throughout acetyl-CoA and glucogenic amino acids enter through TCA intermediates or pyruvate (46).

## Metabolic Regulation of Mucosal Barriers: Epithelial Cells

As the interface separating host and environment, mucosal barriers are key players actively involved in building tolerance to external particles or the development of allergic inflammation. In recent years, it has been shown that metabolic regulation of mucosal barriers is important in allergic responses. Reactive oxygen species (ROS), including hydrogen peroxide (H_2_O_2_), hydroxyl radical (OH), superoxide anion radical (O_2_^–^), and nitric oxide (NO), play an important role in antimicrobial immunity in the mucosal layer. Nevertheless, unchecked production can lead to mucosal barrier damage and dysbiosis, suggesting a new key role in inflammatory lung diseases (47–51). This can be exemplified in chronic rhinosinusitis (CRS), in which a complete metabolic rewiring of epithelial cells has been found. Specifically, ATP metabolic pathways were downregulated while carbohydrate and lipid metabolism pathways were upregulated leading to ROS accumulation. Such metabolic rewiring suggests a long-term inability to keep up with an increased metabolic demand during chronic inflammation (52). When challenged by allergens, barrier cells including bronchial epithelial cells and intestinal epithelial cells can overproduce ROS by up-regulating NADPH oxidase and activating nuclear factor kappa-light-chain-enhancer (NF-κB) signal pathway, leading to elevated IL-1, IL-5, IL-6, IL-33, thymic stromal lymphopoietin (TSLP) (53–56). Furthermore, increased intracellular levels of ROS can also lead to mitochondrial damage, generation of toxic metabolites, and oxidative DNA damage leading to cell apoptosis or autophagy weakening the epithelial barrier and promoting allergen introduction throughout the membrane (57). The allergens itself are also able to induce ROS production. For example, pollen NADPH oxidases increase ROS production leading to recruitment of neutrophils independent of the immune response (54). Not only allergens, but Th2-related cytokines such as IL-13 have been shown to promote ROS production and have been linked to increased inflammation in asthma models (58). Furthermore, superoxide dismutase (SOD), an enzyme with antioxidant protective activity, was found to be impaired in asthma patients compared to control subjects, which triggered apoptosis and shedding of airway epithelial cells and accelerated inflammatory responses (59).

Apart from oxidative homeostasis, glucose and FA metabolism are also pivotal for the maintenance and regeneration of airway epithelium. The proliferation and differentiation of airway epithelial progenitor cells need a moderate level of glucose. The blockade of glucose uptake or glycolysis disrupts airway club cells proliferation while promoting ciliated and goblet cell differentiation, failing to restore the homeostasis of the epithelial layer and promoting asthma chronification (60). Moreover, influenced by peroxisome proliferator-activated receptor gamma (PPAR-γ1, PPAR- γ2) and activator protein 1 (AP-1) transcription factors, glycolysis and OXPHOS were found to be upregulated in the epithelial cells during epithelial-mesenchymal transition (EMT) suggesting a specific metabolic rewiring during tissue remodeling (61). Lipid metabolism plays a key role in inflammation as mediators such as leukotrienes and imbalance in lipid metabolism have been associated with lung inflammation. More specifically, expression of stearoyl-CoA desaturase (SCD), an important enzyme in the synthesis of monounsaturated FAs (MUFAs), has been found to be significantly lower in epithelial cells from asthmatic patients (62). Other enzymes from FA metabolism such as acetyl carboxylase beta (ACC-β) or FA synthase (FAS) also presented distinctive expression patterns in asthmatics, indicating that the imbalance on FA metabolism in asthma patients results in airway hyper-sensitivity and reduced antiviral defense (62).

FA-binding protein 5 (FABP5) is mostly expressed in epidermal cells and plays an important protective role against excessive oxidative damage to lipids in lung infection (63). It has been shown that FABP5 can also help bronchial epithelial cells defend against cigarette smoke exposure and bacterial infection through its interaction with PPARγ (64, 65). Moreover, FABP5 positively regulates the expression of interleukin 1 receptor-like 1 (ST2) – an IL-33 receptor – in alveolar epithelial cells which suppresses excessive activation of ILC2 during allergic inflammation as well as maintaining mucosal barrier homeostasis and its anti-inflammatory function by assisting in retinoic acid (RA) generation (66–70). FABP5 and ST2 were found to be downregulated in the lung tissue of high−fat diet−fed compared to normal diet−fed mice, which might explain why obese people are more susceptible to allergic lung inflammation (70). As such, the positive effects of FABP5 should be investigated further – specifically, which FA are preferably transported.

Some lipid metabolites have a significant influence on allergy responses in mucosal barriers. 12(S)-hydroxyheptadeca-5Z,8E,10E-trienoic acid (12-HHT), one of the metabolites formed during unsaturated FA oxygenation (71), was found to be an endogenous agonist of leukotriene B_4_ receptor type 2 (BLT2) (72). By increasing claudin 4 (CLDN4) expression through the Gαi protein-p38 mitogen-activated protein kinase pathway, the 12-HHT–BLT2 axis is able to maintain epithelial barrier functions and prevent inflammation (73). 15-oxo-eicosatetraenoic acid (15-Oxo-ETE), synthesized by epithelial and MCs metabolism, may increase the dysregulation of arachidonic acid metabolism through the 15-lipooxygenase pathway and the severity of enhanced sinonasal disease observed in aspirin-exacerbated respiratory disease (AERD) (74).

Not only does the metabolic regulation of epithelial cells influence the host response to allergy, but also microbial and xenobiotic metabolites can influence epithelium in allergic responses. The aryl hydrocarbon receptor (AhR) is a ligand-activated transcription factor that regulates metabolization of xenobiotic toxicants by activating genes including phase I xenobiotic-metabolizing enzymes and phase II enzymes, such as NADPH quinone oxidoreductase 1 (75). Bronchial epithelial cells of AhR knockout mice are induced to EMT and become more susceptible to hyperoxic lung damage because of reduced antioxidant enzymes and increased inflammation (76, 77). Furthermore, in a human cohort study, a decreased expression of AhR in human bronchial epithelial cells also led to the worsening of allergic asthma symptoms (76).

In the case of microbial metabolites, short-chain FAs (SCFAs) – mainly butyrate, propionate, and acetate – can be very crucial resources for mucosal cells to provide energy (78) as well as to stimulate epithelial production of RA (79). P-cresol sulfate (PCS), a microbially-generated product of Tyr metabolism in the colonic epithelium (80), was recently found to distally protect the host against allergic airway inflammation such as asthma (81). While CCL-20 plays an important role in DC recruitment in allergy and asthma (82), PCS could bind in the interdomain pocket of epidermal growth factor receptor (EGFR) (83) and block signal transduction of toll-like receptor 4 (TLR-4) to inhibit CCL-20 production by airway epithelial cells and reduce allergic airway responses (81). Lactic acid-producing bacteria can alleviate shrimp tropomyosin-induced allergic mucosal disorders *via* regulation of arginine and proline metabolism and subsequent mammalian target of rapamycin (mTOR) pathway activation (84). All these studies above indicate how immunometabolism in mucosal barriers shapes the inflammatory or allergic responses.

## Metabolic Regulation of Antigen-Presenting Cells: Macrophages and Dendritic Cells

APCs are critical for the initiation of adaptive immune responses and maintenance of peripheral tolerance. They uptake and process antigens which they later present to T cells. DCs are professional APCs contained in the peripheral blood but also DCs are found in tissues such as skin, nose, lungs, stomach, and intestine. DCs remain poorly immunogenic until allergens or pattern recognition receptor (PRR) ligands trigger their activation, a process that is linked to cell metabolism alterations (85). Multiple evidence has shown distinct metabolic requirements of each DC subset and its specific response. While resting or immature DCs obtain ATP by oxidative phosphorylation in mitochondria, TLR-activation rapidly enhances glycolysis, the accumulation of succinate, and the generation of citrate, which is used for the synthesis of FAs and prostaglandins, and to expand membranes in the endoplasmic reticulum and Golgi (86). The TLR-mediated “glycolytic burst” supports DC immune activity by increasing the synthesis and secretion of inflammatory cytokines. Conversely, tolerogenic properties of DCs rely on FAO, as it was observed by using different molecules that favor OXPHOS such as resveratrol, vitamin-D3, and dexamethasone (87, 88) and through the study of tolerogenic human DC metabolism (89). Metabolic control in DCs and monocytes has also been linked to the epigenetic changes and subsequent phenomenon of “trained immunity” – a memory, induced in cells of innate immunity such as DCs and monocytes/macrophages (90). Importantly, this phenomenon has been suggested to play an important role in the induction of tolerance to allergens during allergen-specific immunotherapy (AIT) as tolerogenic plasmacytoid DCs, CD141^+^ myeloid DCs, and intermediate monocytes were increased after 1 year of therapy whereas pro-inflammatory CD1c^+^ myeloid DCs and non-classical monocytes were reduced (91).

Food tolerance is orchestrated by intestinal DCs located in lymphoid tissue and lamina propria of the small intestine and colon (92), where dietary and gut microbiota-derived FAs have a significant influence as it has been reviewed before in detail (93). Polyunsaturated FAs (PUFAs), especially *n*-3 PUFAs, inhibit the pro-inflammatory phenotype of DCs. In contrast, saturated fatty acids (SFAs) increase DC maturation, activation, and T-cell stimulation properties. Moreover, gut bacterial metabolites, such as SCFAs and biogenic amines, enhance DC regulatory activity, leading to the induction of T reg cells and IL-10-secreting T cells (94). Although clinical data show contradictory results, early introduction of fish to diet (*n*-3 PUFAs) reduces sensitization to foods and development of allergic disease in children (95) and prevents allergic sensitization to cow’s milk protein in mice due to a reduction of classical DC type 1 (cDC1) population and an increase in Treg numbers (96). Remarkably, the type of FA ingested not only influences the protective or pro-allergenic role of DCs in food allergy. A higher risk of uncontrolled asthma has been documented in populations with increased consumption of SFAs- and *n*-6 PUFA-rich food, in contrast to a lower incidence of asthma in populations with higher proportions of dietary *n*-3 PUFAs. Similar observations have been made on allergic rhinitis and atopic dermatitis (AD) development in children, while in adults FAs influences are under debate (93, 97).

Another link between metabolism and the regulatory role of DCs in allergy has been confirmed by using rapamycin (mTOR inhibitor) and murine models of mTOR gene depletion. mTOR activation influences DC immune response by stimulating protein synthesis, glycolysis, mitochondrial functions, and lipid synthesis (98). Inhibition of mTORC1 during TLR triggering promotes IL-12 production and inhibits expression of IL-10 and interferon (IFN) type 1 by DCs. A recent study showed the role of the mTOR signaling pathway in the pathogenesis of allergic asthma proving that upon mTOR inactivation, CD11b^+^ DCs of the lung can skew allergic inflammation from eosinophilic Th2 to neutrophilic Th17 polarity. The mechanism for this change is dependent on DC IL-23 production and an increase in the use of FAO (99).

**Figure 3:**
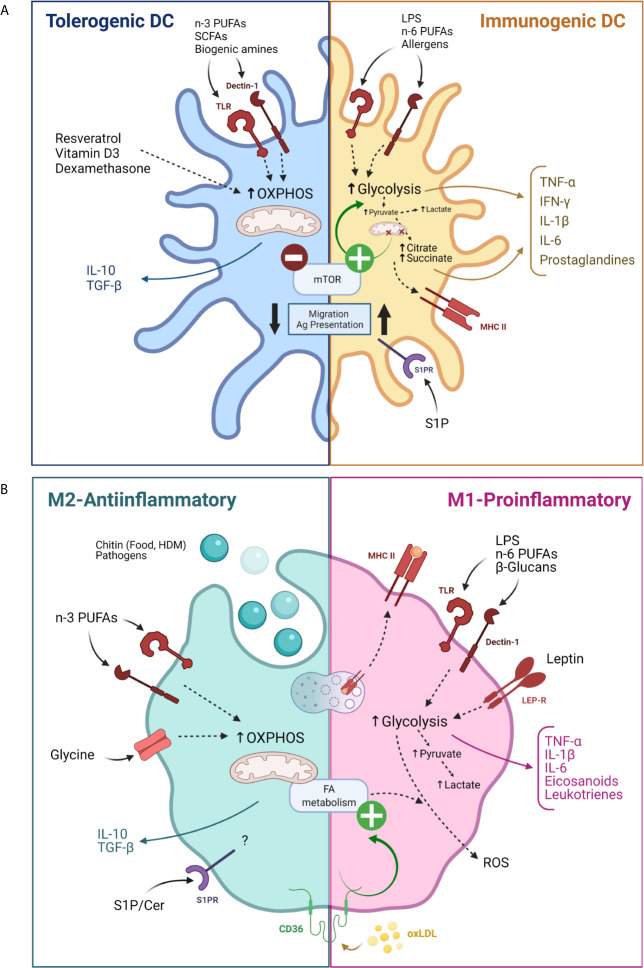
Modulation of antigen presenting cells activity by their metabolism. **(A)** Modulation of dendritic cells by their metabolism for tolerogenic (blue) and pro-inflammatory (yellow) functions. **(B)** Modulation of macrophages by their metabolism for M2 (green) or M1 (pink) differentiation. Cer, Ceramide; FA, Fatty acid; HDM, House dust mite; LEP-R, Leptin receptor; MHC, Major histocompatibility complex; oxLDL, Oxidized low density lipoprotein OXPHOS, Oxidative phosphorylation; PUFAs, Poly unsaturated fatty acids; ROS, Reactive oxygen species; SCFAs, Short chain fatty acids; S1P, Sphingosine 1 phosphate; SP1R, Sphingosine 1 phosphate receptor. Created with BioRender.com.

Additionally, sphingolipids are involved in DC maturation, activation and migration. It has been shown that blocking sphingosine 1-phosphate (S1P) receptors reduces the migration and the antigen presentation capacity of DCs, which decreased allergic asthma in mice (100). In addition, treatment with a non-phosphorylated S1P analog - 2-Amino-2-[2-(4-octyl-phenyl)-ethyl]-propane-1,3-diol hydrochloride (FTY720) - reduces *in vitro* and *in vivo* allergic respiratory features (101–103). A dysregulation of skin-S1P homeostasis has been discussed in the pathogenesis of AD. Indeed, topical application of S1P diminished DC antigen capture and abrogated DC migration which improved AD treatment (103, 104). Similarly, the topical administration of S1P in a murine contact hypersensitivity model reduced the number of DCs within the lymph node and decreased IL-6 and IFN-γ secretion (105). A summary of the aforementioned DC metabolism modulation can be found in **Figure 3A**.

Macrophages respond to a variety of stimuli and can display a continuum of functional states that have been broadly classified between two functionally polarized extremes: M1 or pro-inflammatory macrophages and M2 or anti-inflammatory macrophages, each one associated with specific gene, protein, lipid signatures and metabolism requirements. Briefly, M1 macrophage functions mainly rely on aerobic glycolysis and succinate and citrate accumulation, while M2 macrophages properties depend on OXPHOS, enhanced FAO and a decreased glycolysis (106). Macrophage polarization can be redirected by modulation of metabolism *in vitro* (107).

Macrophages influence the response of allergen-specific T CD4^+^ helper as well as T CD8^+^ cytotoxic lymphocytes. Besides, murine macrophages produce histamine-releasing factor, which stimulates histamine release and IL-4 and IL-13 production from IgE-sensitized basophils and MCs (108).

Macrophages located at the oral and gut mucosa work as scavengers of damaged tissue and foreign material that is processed and presented to T cells. They also produce cytokines and chemokines that stimulate other cells such as fibroblasts (109, 110). In mice, oral and small intestine macrophages are key cells for tolerance as they exhibit an anti-inflammatory gene signature and produce IL-10, which maintains Treg activation (111, 112). Activation of macrophages *via* TLR-4 by a wide range of molecules is one of the main stimuli changing macrophage metabolism, function, and polarization towards M1-phenotype (113, 114). *Via* TLR-4 macrophages produce the typical M1-cytokines and lipid mediators such as PGD_2_, prostaglandin E_2_ (PGE_2_), and their metabolites (115). In addition, activation of the NLR family pyrin domain containing 3 (NLRP3) and other inflammasomes with subsequent IL-1β, IL-18, and pyroptosis, are tightly regulated by various metabolic cues (116). For example, PGE_2_, released by macrophages in response to TLR-4 activation and following initiation of arachidonic acid metabolism, limits macrophage function by inhibition of NLRP3 inflammasome activation (114).

Specific food-derived metabolites can also activate macrophages, such as the polysaccharide chitin, which is found in the cell walls of bacteria and fungi, mushrooms, and the exoskeleton of crustaceans and insects (117–119). Macrophages recognize chitin fragments through PRRs, mainly TLR-2 and Dectin-1, and, as consequence, they produce cytokines involved in inflammation and allergic responses. Also, macrophages of the lung and digestive tracts constitutively display chitinolytic enzymes which are increased in allergic conditions (120). Chitinase 3-like 1 (CHI3L1) protein levels are significantly higher in children with food allergy and ovalbumin (OVA)-sensitized mice than in healthy controls, and this has been linked to M2 macrophage polarization (121). Moreover, some chitinases from plant origin are a well-known group of food allergens (122). Another metabolite that modifies macrophage response is the amino acid Gly, which might prevent acute allergic response in cow’s milk allergy through the inhibition of macrophage inflammatory response (123). Additionally, ingested β-glucans bind to PRRs on macrophages and stimulate IL-12 and TNF-α cytokine production, which inhibits intestinal Th2-dependent allergies (124).

Several studies have demonstrated that airway macrophages (AMs) display altered metabolism in respiratory allergies, mainly *via* dysfunction in eicosanoid, glycolysis, and FA pathways (125). AMs degrade allergens through the secretion of specific enzymes, like chitinase, which neutralizes chitin (117). M1 and M2 may coexist in healthy and asthmatic lungs (126). Alveolar macrophages adapt their metabolism according to the required function. They utilize aerobic glycolysis to rapidly generate cytokines and ROS for pathogen defense but employ mitochondrial respiration to fuel inflammatory responses. Both macrophage subtypes are increased in asthmatic patients. M2 release IL-10 and IL-13, which promotes Th2 lymphocytes differentiation, as well as eosinophil recruitment (127). Likewise, they sustain and enhance inflammation initiated by Th2 cells, ILC2, eosinophils, and basophils (128). Mouse models suggest that M1 macrophages are beneficial in asthma by decreasing the recruitment of immune cells and inducing a pro-tolerogenic response. However, M1-associated features such as metabolic stress (ROS) and IFN‐γ and TNF‐α proteins are elevated in bronchoalveolar lavage and AMs of asthmatics patients (125, 129). These changes have also been related to an imbalance between oxidized and reduced forms of glutathione in AM (130). Additionally, increased production of the eicosanoid 5-hydroxyeicosatetraenoic acid (5- HETE) and leukotrienes B4 (LTB4) and E4 (LTE4) has been detected in AMs from asthmatic patients stimulated *ex vivo* (131). An interesting link between metabolism influence on macrophage functions in allergy is observed in obese patients with asthma, where there is a shift from M2 to M1 partly due to increased *n*-6 PUFAs and leptin levels found on these subjects (132). Both molecules trigger pro-inflammatory signaling pathways on macrophages and are involved in murine models of allergic inflammation (133–135).

Macrophage metabolic deregulation also plays a role in cutaneous allergic reactions. They accumulate in AD inflamed lesions where they interact closely with inflammatory immune cells, leading to the aggressive progression of severe AD (136). In lesional skin of AD patients, macrophages express higher levels of CD36^+^ than in normal skin (137), which is an M2-associated marker responsible for FA importation but whose signaling under dysregulated FA metabolism drives chronic inflammation (138). In contact dermatitis, macrophages present haptens to Th1 lymphocytes, which in turn produce IFN-γ that activates macrophage oxidative metabolism inducing skin damage (139). A summary of the aforementioned macrophage metabolism modulation can be found in **Figure 3B**.

Hence, metabolic alterations associated with allergic disorders impact APC immunomodulatory and inflammatory properties. In addition, allergens, PRR-ligands, cytokines, and lipids involved in allergic reactions affect the intracellular metabolism of APCs, determining their immunogenic or tolerogenic functions.

## Metabolic Regulation of CD4 T Cells: Th and Treg

T lymphocytes are the essential part of the adaptive immune response. T cells can be divided into CD4^+^ and CD8^+^ populations, which have different functions. CD8^+^ cells constitute part of the immediate and memory cellular cytotoxic response in cancer and infectious diseases, whereas CD4^+^ cells are the main orchestrators of the adaptive immune response and antibody production. In allergic diseases, two subpopulations of CD4^+^ cells play major roles: Th2 cells, which express transcription factor GATA binding protein 3 (GATA3) and induce the production of IgE, and regulatory T cells (Tregs) which are the main inducers of tolerance towards the antigens. In the past years, it has been shown that the metabolic reprogramming of these cells has a critical influence on their function.

Th subsets rely mainly on glycolysis to quickly achieve great amounts of energy during a short span after activation (35, 37, 140, 141) whereas Tregs rely on OXPHOS as the energy supplier pathway to obtain a constant energy supply over longer periods of time (142, 143). Often, they use FA to fuel OXPHOS however the use of long-chain FAO to produce energy in any type of T cell has been shown to not be linked to their function (144).

An important requirement for T cell survival is IL-2. Oesterich et al. demonstrated that IL-2 might regulate CD4^+^ survival partially by regulating their metabolism. They showed that transcription factor B cell lymphoma 6 protein (Bcl-6) inhibited the expression of several glucose receptors and key enzymes from the glycolysis pathway such as HK2 and PKM2. This transcription factor was active only in a low IL-2 expression setting and was counteracted by Th1 transcription factor T-box transcription factor 21 (T-bet) (145). Moreover, it has been shown that when activated T cells are provided with co-stimulation and growth factors but are blocked from engaging glycolysis, their ability to produce IFN-β is markedly compromised (146). The importance of Bcl-6 for glycolysis was also shown *in vitro* and in a graft *vs* host disease (GVHD) mice animal model in which inhibition of glucose uptake led to preferential Treg differentiation instead of Th1 or Th17 (147). This differentiation could be a consequence of the accumulation of glycolysis intermediate subproduct PEP, which inhibits the reabsorption of intracellular calcium to the endoplasmic reticulum (ER) by suppressing the transporter sarcoendoplasmic reticulum Ca2+-ATPase (SERCA) and thus making T cells more prone to activation (148). At the same time, glycolysis end-product lactate is able to modify T cell phenotype to Th17 in chronic inflammatory diseases by inhibiting the use of glycolysis to create pyruvate and activating the PPP. This process switches the uses of NADPH and citrate for FA synthesis which promotes phosphorylation of STAT3 and Th17 transcription factor RORyt (149). ACC1 plays a crucial role in this switch to the Th17 phenotype as its inhibition leads to blocking the glycolytic-lipogenic pathway and promotes differentiation to a Treg phenotype instead (150).

In the case of Treg cells, it has been recently shown that TLR ligands increase PI(3)K-Akt-mTORC1 signaling, glycolysis, and expression of GLUT1, impairing their suppressive functions (151). Moreover, airway Th2 cells which mediate allergic responses against house dust mite (HDM) have been shown to also rely on both glycolysis and lipid metabolism (152). Additionally, HIF-1α is a key factor to regulate T cell responses (152). Its deletion leads to an increase in Treg immunosuppression in the tumor microenvironment and inhibits their migratory capacities (153). Furthermore, complete loss of HIF-1α has been proved to lower the humoral response after carrier immunization and Th cytokine production, suggesting it is a possible target for allergen immunotherapy (154). Nevertheless, this process appears to differ in the gut as HIF-1α expression increased the number of Treg and their inhibitory functions in a colitis mouse model (155). As such, the intervention might not be effective in food allergy AIT.

Programmed cell death 1 (PD-1) and its ligand (PD1L) are key proteins in the control of immune responses and T cell fate (156–158). Patsoukis et al. have shown that PD-1 activation in T cells profoundly changes their metabolism by inhibiting glycolysis and glutaminolysis to promote FAO (159). At the same time, Palaskas et al. showed as well in a similar model an inhibition of *de novo* nucleoside phosphate synthesis which limits the proliferation of these cells. This phenotype could be replicated by inhibiting mTOR (160). These studies suggest new effects of the PD-1 and PDL1 axis to control inflammatory responses and could lead to the use of metabolic treatments to achieve immune suppression and tolerance as shown in allograft animal models (161).

Recent studies have shown that mitochondrial respiration and stability are also important for naïve T cells, which initiate mitochondrial biogenesis upon activation and undergo the synthesis of *de novo* nucleic acids using SHMT2 (162). Upon its knockdown, T cells engage in apoptosis. Milasta et al. observed that conditional ablation of mitochondrial interspace protein AIF in T cells inhibited their function. AIF did not impact the development nor increase cell death (163).

**Figure 4:**
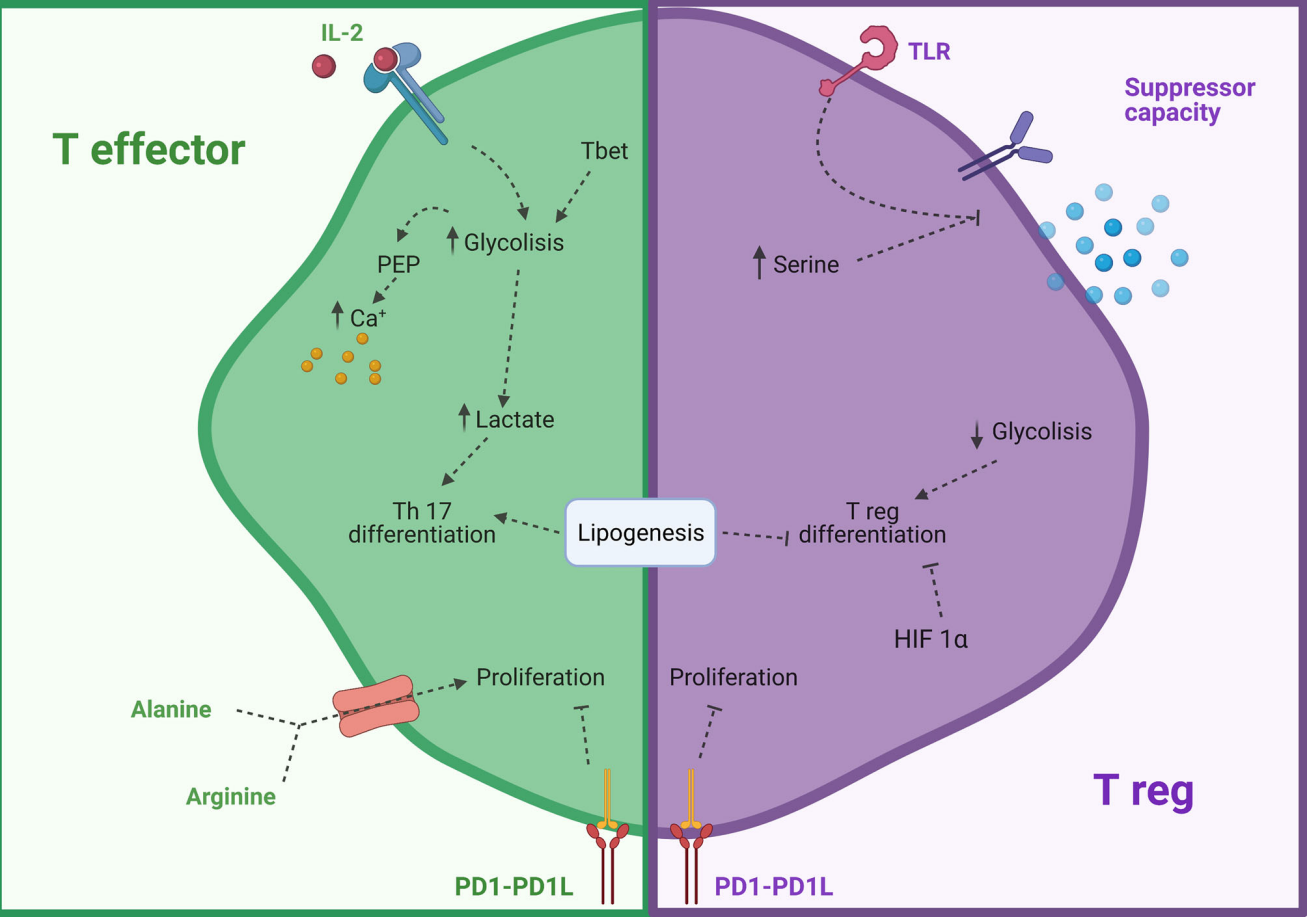
Modulation of CD4 T cells by their metabolism. In green modulation of T effector cells and in purple modulation of T reg cells. HIF 1β, Hypoxia inducible factor 1 β; PD-1, Programmed cell death protein 1; PD1L, Programmed cell death- ligand 1; PEP, Phosphoenol pyruvate; TLR, Toll-like receptor. Created with BioRender.com.

In addition, amino acids can potently control T cell functions. Arginine (Arg), an amino acid linked to NO production, is increased in asthmatic individuals (164, 165), and can influence in great manner T cells as a high intracellular concentration of Arg enhances T cell survival and function (166). Moreover, extracellular vesicles containing catabolic enzyme arginase-1 suppressed proliferation of both CD4^+^ and CD8^+^ cells, signaling the importance of this amino acid in T cell responses and suggesting a new potential drug target in asthmatic allergic diseases (167). Alanine (Ala) is also a key factor to exit quiescent state in naïve and restimulated T cells. Its deprivation leads to impaired functions, as activated T cells upregulate the expression of serine transporter and rely on this amino acid for protein biosynthesis (168). Another neutral amino acid with important functions is serine, which is required for correct glutathione metabolism and Treg suppressive functions (169, 170). Specific deletion of glutamate-cysteine ligase, a key enzyme in the biosynthesis of glutathione, led to an increase in intracellular serine, loss of expression of Foxp3, suppressive function, and increased mTOR signaling (169). A summary of the aforementioned metabolic reprogramming T cells can be found in **Figure 4**.

Due to the importance of immunometabolism in T cell differentiation and Treg function, further studies in the context of allergic inflammation are needed to explore its potential and translate it to new therapies.

## Alterations of Energy Metabolism in Inflammatory Pathologies

Metabolic reprogramming of immune cells plays a pivotal role in different pathologies such as cancer, type 2 diabetes (T2D), obesity, and infectious diseases (171–175).

The ability of metabolic rewiring of tumor cells to support their growth is a hallmark of cancer and has been well known since the beginning of the XX^th^ century, when the Warburg effect was initially discovered in tumoral cells (35, 36, 176). However, the Warburg effect has also been described in other cell types such as activated immune cells, signaling that metabolism may be extrapolated between different conditions or diseases (35, 36, 177–179). It is well known that tumoral cells modulate and escape the surveillance of immune cells surrounding them in the tumor microenvironment and in some cases by modifying their metabolism. For example, tumor-associated macrophages (TAMs) are abundant in most malignant tumor microenvironments and have specific immunological roles in cancer (180, 181). In a mouse model, lactic acid produced by tumoral cells could polarize TAMs towards an M2-like phenotype by a mechanism that depends on transcription factor HIF1α promoting cancer progression (182). Additionally, DCs activation depends on the use of citrate synthesized from pyruvate as a substrate for lipogenesis (86). Thus, lactate secretion by tumoral cells might also inhibit cross-presentation to T cells leading to immune evasion.

Other metabolites such as Arg also have been found to play a crucial role in the polarization between TAMs with an M1- or M2-like phenotype (TAM1 and TAM2, respectively). While TAM1s express inducible nitrate oxidase synthase (iNOS) producing high quantities of cytotoxic nitric oxide (NO) after stimulation by pro-inflammatory cytokines: TAM2s express arginase 1, which converts Arg to ornithine, promoting M2 polarization surrounding cancer cells (183). Competition for glucose between cancer cells and T cells has been shown to be critical in terms of determining whether tumors progress or regress (184). Following activation, T cells need to grow rapidly, proliferate, and generate cytokines to direct a functional immune response and thus, a metabolic switch is required (140, 141). Therefore, upon activation, the metabolic state of T cells resembles that of cancer cells. However, in the tumor microenvironment, low concentrations of glucose and direct immune suppression by the tumoral cells inhibit T cell activation (185).

Trp metabolism is also a key player in immune responses and cancer (186). Kynurenine, a metabolite derived from Trp, plays an important role in cancer, as it is absorbed by myeloid-derived suppressor cells (MDSCs) and inhibits the function of CD8^+^ T cells (187, 188). Kynurenine and other potential catabolic metabolites are endogenous ligands for AhR, a transcription factor that broadly modulates immunity. The activation of AhR by kynurenine can induce forkhead box p3 (Foxp3) expression in naïve T cells and prevent Th17 maturation, causing proliferation of Tregs (189, 190). Moreover, Trp depletion inhibits T cell proliferation, and indoleamine 2,3-dioxygenase 1 (IDO1) facilitates tumor evasion under immune surveillance. Additionally, activated T cells and B cells are found to possess increased Gln uptake, and Gln metabolism is critical for controlling reactive oxygen species (ROS) levels and for maintaining the balance between effector T cells (Teff) and Treg cells (191). Depletion of this metabolite blocks the proliferation and cytokine production of T cells (150). Recent studies have reported that Ser metabolism-related enzymes are induced upon naïve T cell activation and required for proliferation and survival. Specifically, they are required for *de novo* nucleotide biosynthesis (162, 192). Additionally, lipid metabolites such as cholesterol and prostaglandin E_2_ (PGE_2_) are extensively involved in the activation and function of immune cells (193).

Several studies have demonstrated that overnutrition is associated with low-grade, chronic inflammation increasing the risk of metabolic and cardiovascular disease and promoting autoreactivity (185). Specifically in T2D, the development of insulin resistance involves alterations of energy metabolism and inflammatory signals derived from immune cells (194). Furthermore, numerous studies have determined that T2D impairs the host defenses against pathogens including suppression of cytokine production, phagocytosis impairment, immune cells dysfunction, and incapacity to eradicate microorganisms (195, 196). Studies in mice models have shown that CD8^+^ T cell activation is one of the earliest events in the inflammatory response to obesity, preceding M1-like macrophage activation/infiltration in the adipose tissue (197). This effect is enhanced when elevated SFA levels are combined with hyperglycemia, as this combination appears to cause additive effects on β- cell proliferation (135). Finally, high quantities of ceramides and sphingomyelins in serum in diabetic patients induce inflammasome NLPR3 dependent activation of macrophages and increased IL-1β (198). Due to the impact of those molecules in asthmatic disease (114, 199–201), ceramides and sphingomyelins could be a potential biomarker for disease prognosis in allergic asthma.

Obesity is a co-morbidity characterized by higher levels of basal inflammatory state and associated with more severe asthma (202–204). This can be exemplified by a switch from an anti-inflammatory M2 phenotype to a pro-inflammatory M1 activation state of adipose tissue macrophages (ATM) during weight gain. Nonetheless, other studies have highlighted the heterogeneity and plasticity of ATM phenotypes in response to different metabolic stimuli such as high glucose or high insulin (205, 206). This plasticity is not limited to ATM, as alveolar macrophages in high insulin environments also showed defects in phagocytosis activity, highlighting the importance T2D might have on lung respiratory diseases (207).

In obesity, hypercholesterolemia leads to cholesterol accumulation in macrophages and other immune cells. The accumulation promotes inflammatory responses, including augmentation of TLR signaling, inflammasome activation, and the production of monocytes and neutrophils in the bone marrow and spleen (208–210). Inflammation in visceral adipose tissue (VAT) is a major driver of insulin resistance (211–213). Exposure of macrophages to very-low-density lipoproteins (VLDL) and SFAs promotes an M1-like phenotype by stimulating the secretion of proinflammatory cytokines (214, 215). Free FAs promote inflammation of proinflammatory macrophages in obese adipose tissue. These macrophages can also be distinguished from classically activated macrophages by their higher expression of genes involved in lipid metabolism (216). Obesity is also associated with reduced levels of Tregs (217).

In infection, a successful immune response relies on the ability of T cells not only to proliferate extensively and attain effector functions but also to form long-lived memory T cells that can respond again to future antigen encounters. FAO engagement is critical for the generation of memory CD8^+^ T cells, while mTOR signals dampen this development (218, 219). Memory CD8^+^ T cells possess a higher mitochondrial spare respiratory capacity than CD8^+^ Teffs and have greater mitochondrial mass than naive or activated T cells (220). Lipids such as FAs regulate inflammatory processes and are signaling molecules for the activation and function of macrophages, invariant Natural Killer T (iNKT) cells, and T cells (221). Moreover, mucosal-associated invariant T cells (MAIT) in obese patients present a defective mTOR functionality which leads to defective glycolysis and anti-microbial defense which could play a factor in the proinflammatory gut environment in these patients (222).

Finally, the interaction between microorganisms and their products with the immune cells can also modulate the immunometabolism. For example, antibiotic treatment promoted the overgrowth of *Candida* species in the gut of a mice model. This overgrowth resulted in increased levels of PGE_2_ in plasma which shifted macrophage polarization to M2 in the lung and thus enhancing susceptibility to allergic airway inflammation (223). Microbial products also play a role in immune system modulation as microbially-derived SCFAs can promote hematopoiesis and alter the gene expression profile of local macrophages (224, 225). Moreover, butyrate stimulates CD103^+^ DCs to produce higher levels of transforming growth factor-beta (TGF-β) which in turn promotes Treg differentiation (226). RA and dietary fiber also promote these CD103^+^ DC–Treg interactions (227). Finally, tissue-resident memory T (TRM) cells are key players to protect epithelial barrier tissues against pathogens (228). Mouse CD8^+^ TRM cells generated by viral infection differentially express high levels of several lipid uptake and intracellular transport molecules, including FABP4 and FABP5. The persistence of these CD8^+^ TRM cells in the skin was strongly diminished by inhibition of mitochondrial free FAO *in vivo*, suggesting that FABP4 and FABP5 have a critical role in the maintenance, longevity, and function of CD8^+^ TRM cells. As such, CD8+ TRM cells require the consumption of exogenous free FAs and their oxidative metabolism to persist in tissue and to mediate protective immunity (229).

## Techniques Employed in the Study of Immune Metabolism

In recent years different techniques have been used or adapted to study immunometabolism with a specific increase in the use of -omic techniques as they allow the acquisition and analysis of high amounts of data from the different levels of systems biology. The most used in immunology in recent years are genomics and transcriptomics. In the first sequencing studies, metabolic traits were used to define phenotypes (230). Due to the extensive databases, single-cell resolution, and broad applications, transcriptomics is a very popular technique, especially since the development of functional enrichment analysis which allowed to identify more genes and pathways related to cell metabolism. One such example is the study by Stevens et al. (74) who utilized single-cell RNA sequencing to compare the transcriptional profile of cells from patients with AERD and chronic rhinosinusitis with nasal polyps (CRSwNP) and could identify a novel lipid mediator,15-oxoicosatetraenoic acid (15-Oxo-ETE). Moreover, this technology can be used to obtain information about the impact of microbial metabolism as proposed by Singer et al. (231), who performed single-molecule real-time sequencing technology to identify phylogenetic microbial communities in high-resolution, enabling them to profile them and predict their metabolic potential.

Proteomics has also been extensively used in immunometabolism research. Proteomics is based on studying the proteic compartment of living cells. It provides more information than transcriptomics, since the key components controlling cellular metabolism are enzymes, and as such, their regulation can be done by post-translational modifications. Several studies have used proteomics for the study of immunometabolism. Tan et al. used it to study all the metabolic effects that are triggered on T cells upon TCR activation, from which OXPHOS was found to be critical for the T cells to proliferate and differentiate (232). Ron Harel et al. also showed that mitochondrial one-carbon metabolism was also crucial for T cell activation, survival, and adequate OXPHOS energy output (162).

The last -omic technique to investigate immunometabolism is metabolomics, the science advocated to the study of the metabolism in living organisms (233). Metabolomics enables the characterization of metabolites (endogenous small molecules) that are the intermediate or final products of biochemical reactions, revealing connections among different pathways that operate within a living cell (234). Currently, there is no single technique that detects the entire metabolome, as the metabolite repertoire encompasses wide concentration ranges and different physicochemical properties. However, high-throughput analytical techniques – such as mass spectrometry (MS) and nuclear magnetic resonance spectroscopy (NMR) – allow to detect high numbers of metabolites at the same time and give the structural information necessary for identification. Additionally, separation techniques – such as liquid chromatography, gas chromatography, and capillary electrophoresis – have been coupled with MS to increase the number of metabolites detected (235), and give complementary information. Metabolomics can be applied to any biological sample such as plasma, cells, or feces. This science can work following two approaches, non-targeted and targeted analysis. In the first one, the aim is to detect as many metabolites as possible in a single analysis in each sample, selecting afterward those statistically different metabolites between groups. This approach is hypothesis-free and leads to the discovery of promising metabolic changes to better understand the molecular mechanisms in the pathology. On the other hand, in targeted analysis, specific metabolites are selected based on previous knowledge, analyzed and, frequently, quantified. Usually, targeted analysis is applied to confirm and validate previous findings from the non-targeted analysis. These findings can constitute, after a proper validation, potential biomarkers used in the clinic for diagnosis or prognosis.

Immune cells have been analyzed by metabolomics. In a recent study, the characterization of cell subpopulations of mouse peritoneal macrophages after fluorescence-activated cell sorting (FACS) was carried out. The authors observed that FACS-treated cells have plasma membrane-derived metabolites causing inflammation, cell damage, stress, and specific changes in energy consumption-related metabolites (236). Additionally, metabolomics has also been used to characterize the metabolic profile of allergic patients, in which severe patients presented a characteristic pro-inflammatory profile (12). Furthermore, isotope tracing analysis using isotope-labeled metabolites – such as glucose or amino acids –can shed light on the flux of the metabolite inside specific pathways (237). These techniques can be used in combination with other omics for a full comprehensive study of the metabolism (166).

Finally, non -omic techniques can also be employed to study immunometabolism. Flow cytometry, a gold standard technique in immune phenotyping, has been recently used to study different metabolic proteins in immune cells at the single-cell level (238). This technique may be a good first step before approaching an -omic technique in which complex technical knowledge might be required. Another popular technique is the use of Seahorse extracellular flux analyses. These *in vitro* studies are based on the real-time measurement of different physical properties in the cell media to reflect the cellular metabolic activity – specifically, oxygen consumption to measure OXPHOS activity and medium acidification for glycolysis. As such, it allows to analyze the effects of different stimuli at the metabolic level. Numerous studies mentioned throughout this review have applied this technique (6, 86, 166, 184, 220).

All of these are the most extensively used techniques in immunometabolism and should give a good snapshot for new researchers into the field. A short summary of the techniques mentioned can be found in **Table 1**.

**Table 1:** Current approaches to study cell metabolism.

Approach	Description	Techniques	Type of sample
Sequencing	Study of all the genetic material present in a sample. Can be performed in DNA (genomics, epigenomics) or RNA (transcriptomics)	NGS: For studying long sequences of human or microbial origin (202)	Biopsy, cells, swabs and stools. Some techniques allow resolution at the single cell level
		Microarray: Study of a specific set of genes/transcripts (12)	
		RNA-seq: To study the whole the transcriptome (239)	
		Chip-Seq/DNA methylation: To study epigenetic changes (240)	
Proteomics	Study of all the protein material in a biological sample. Can be done for a selected few (targeted) or all proteins present (untargeted)	Mass spectrometry: Can be used for both targeted and untargeted studies (241)	Any biological fluid (saliva, serum, plasma), biopsy or cells.
		Protein arrays: Analyzes specific sets of proteins (242)	
Metabolomics	Study of all the metabolites in a biological sample. It can be done for a selected few (targeted) (235) or all metabolites present (untargeted) (243, 244)	LC-MS: metabolites of all polarities (245)	Any biological fluid [saliva (246), serum (247), plasma, urine (248)], biopsy (249) or cells (250)
		GC-MS: volatile metabolites or those that are volatile after chemical derivatization (251)	
		CE-MS: polar metabolites (252)	
		NMR: abundant metabolites (253)	
Flow cytometry	Study of a specific set of proteins	Flow cytometry (238)	Any type of cell. Can be combined with other techniques for the same sample
Extracellular flux analysis	*In vitro* study of the energy metabolism of live cells	Seahorse flux analysis (166)	Any mammalian cell that can be cultured

LC-MS, liquid chromatography coupled to mass spectrometry; GC-MS, gas chromatography coupled to mass spectrometry; CE-MS, capillary electrophoresis coupled to mass spectrometry; NMR, Nuclear Magnetic Resonance spectroscopy; NGS, next generation sequencing.

## Discussion

Cell metabolism actively participates in shaping immune responses in health and disease. Metabolic changes influence immune responses in various disorders, such as obesity, T2D, cancer, infectious diseases, and allergy. Metabolic dysregulation affects all cells in the body, with epithelium, macrophages, DCs, and T cells playing pivotal roles. Changes in metabolic profiles of immune cells affect the balance between immunogenic and tolerogenic phenotypes, subsequently participating in disease pathomechanism. The metabolic status of an individual is closely linked to nutrients availability. Excessive intake of SFAs might increase inflammation, especially in the presence of pre-activated immune cells related to the pathomechanism of the underlying disease. On the opposite, a diet rich in unsaturated n-3 FAs reduced the frequency of allergic diseases highlighting the importance of metabolism and diet in allergic diseases (254). Additionally, dietary habits also influence the metabolism of gut microbiota, which subsequently affects the health status of the host. Host–microbiota crosstalk modulates allergic inflammation not only in the gut but also in peripheral tissues, such as the lungs (224). The influence of metabolism on immune homeostasis in allergic diseases is not fully understood. However, a growing body of evidence suggests the pivotal role of immunometabolism in the regulation of mucosal barriers, altered pathways in antigen-presenting cells, and T cells in allergy. Understanding the complex effects of metabolism on immunological homeostasis is crucial for the prevention and treatment of allergic diseases. Metabolites are a direct link between the metabolic status of an individual and disease phenotype. Detection of metabolites is possible in various samples such as immune cells, serum, plasma, saliva, urine, stool, or tissue biopsies using metabolomics. The non-targeted analysis approach is the most used in exploratory studies because it can highlight the metabolic pathways involved under a condition. Complementarily, the targeted analysis is capable of validating the findings from the previous approach and thus lead to the determination of a more reliable biomarkers for a condition.

Regarding the coverage of metabolites, great efforts have been made to create robust methodologies to cover polar and lipid metabolites independently. In this sense, while analysis of TCA and glycolysis metabolites are a key part of the cellular metabolism, lipids (apart from other roles) are critical regulators of inflammation (255–257). Different studies have demonstrated the importance of lipid mediators in the development of allergy and other related diseases (258). In cells, lipids are participants in cell signaling events and have been associated with immune phenomena such as degranulation, chemotaxis, and sensitization (259). For example, leukotrienes are a class of immune-modulating eicosanoids that have emerged as useful clinical targets for the treatment of allergic diseases (260–262). Another class of lipids is sphingolipids, specifically sphingosine-1 phosphate, which has been closely linked to asthma and allergy progression (12). Other mechanisms such as fatty acid oxidation (FAO) have been observed increased in allergic airway inflammation in immune cells to support the production of cytokines, chemokines, and other factors important in the development of asthma (263).

The relevance of the omic sciences in the clinic relies on the idea that understanding the molecular mechanisms is crucial to improve diagnosis, prognosis, and therapeutic personalized medicine strategies.

Complementarily, several dedicated methodologies such as Seahorse flux analysis assays allow in-depth investigation of the metabolic status. All metabolomic approaches coupled with proteomics, genomics, transcriptomics, and epigenomics will allow for a better understanding of metabolism in allergic diseases.

## Conclusions

The metabolism of immune cells is intimately linked to their differentiation and function. Immune cells under specific microenvironment changes display different metabolic responses. This metabolic reprogramming can be initiated not only by nutrient conditions but also by disease-related molecules –such as inflammatory molecules, pathogens, or allergens. In the present day, ongoing research is focused on understanding the immune cell metabolism in different conditions such as inflammation, cancer, obesity, T2D infectious diseases, and allergy, which could shed light on new therapeutic interventions.

## Author Contributions

JR-C contributed to parts 3, 6, 8, 9. AV contributed to parts 7, 8, and 10. EI contributed to part 5. MH contributed to parts 4 and 8. TB-T contributed to part 2, to figure 3 and to English proof correction. UR contributed to part 8 and 9. DB and MS contributed to parts 1, 9, and 10 and coordinated the manuscript. All authors contributed to the article and approved the submitted version.

## Funding

This work was supported by Instituto de Salud Carlos III (project numbers PI19/00044 and PI18/01467) co-funded by European Regional Development Fund “Investing in your future” for the thematic network and co-operative research centers ARADyAL RD16/0006/0015 and by the Swiss National Science Foundation (SNF) grant nr 310030_189334 (to MS lab), and GlaxoSmithKline (GSK) scientific research grant (to MS lab). JR-C was supported by FPI-CEU predoctoral fellowship and a SEMP fellowship from University of Zurich. TB-T was supported by FPI-CEU predoctoral fellowship. AV is funded by a postdoctoral research fellowship from ARADyAL. MH and UR were supported by the SNF, GSK and SIAF.

## Conflict of Interest

The authors declare that the research was conducted in the absence of any commercial or financial relationships that could be construed as a potential conflict of interest.

## Publisher’s Note

All claims expressed in this article are solely those of the authors and do not necessarily represent those of their affiliated organizations, or those of the publisher, the editors and the reviewers. Any product that may be evaluated in this article, or claim that may be made by its manufacturer, is not guaranteed or endorsed by the publisher.

## Glossary

**Table:**

12-HHT	12(S)-hydroxyheptadeca-5Z,8E,10E-trienoic acid
15-Oxo-ETE	15-oxo-eicosatetraenoic acid
ACC	acetyl carboxylase
acetyl-CoA	acetyl coenzyme A
AD	atopic dermatitis
AERD	aspirin-exacerbated respiratory disease
AHR	aryl hydrocarbon receptor
AIF	Apoptosis inducing factor
AMP	adenosine mono phosphate
AMs	airways macrophages
AP1	Activator protein 1
APCs	antigen presenting cells
ATM	adipose tissue macrophages
ATP	adenosine 5’-triphosphate
Bcl-6	B cell lymphoma 6 protein
BLT2	Leukotriene B4 receptor
CCL2	chemokine (C-C motif) ligand 2
CCL20	chemokine (C-C motif) ligand 2
cDC	conventional DC
CHI3L1	Chitinase 3-like 1
CLDN4	Claudin 4
CRS	chronic rhinosinusitis
CRSwNP	chronic rhinosinusitis with nasal polyps
CysLT	cysteinyl leukotrienes
DAMPs	Damage-associated molecular patterns
DCs	dendritic cells
EGFR	epidermal growth factor receptor
EMT	epithelial-mesenchymal transition
ER	Endoplasmic reticulum
FABP	fatty-acid-binding proteins
FACS	fluorescence-activated cell sorting
FAO	fatty acid oxidation
FAs	fatty acids
FAS	fatty acid synthase
FBP	fructose 1,6 bisphosphate
FcϵRI	high-affinity Fc epsilon receptor I
Foxp3	forkhead box p3
FTY720	2-Amino-2-[2-(4-octyl-phenyl)-ethyl]-propane-1,3-diol hydrochloride
GATA3	Gata binding protein 3
GLUT	glucose transporters
GVHD	graft vs host disease
HDM	House dust mite
HIF1α	hypoxia-inducible factor 1α
HK	hexokinase
IDO1	indoleamine 2,3-dioxygense 1
IFN-γ	Interferon gamma
IgE	immunoglobulin E
IL	interleukin
ILCs	type 2 innate lymphoid cells
Ile	isoleucine
iNKT	invariant Natural Killer T
iNOS	inducible nitrate oxidase synthase
Leu	leucine
LTB^4^	leukotriene B4
LTE^4^	leukotriene E4
Lys	lysine
MAIT	mucosal-associated invariant T cells
MCs	mast cells
MHC	Major histocompatibility complex
MDSC	myeloid derived suppressor cell
moDCs	monocyte-derived DCs
MS	mass spectrometry
mTOR	mammalian target of rapamycin
MUFAs	monounsaturated fatty acids
NADH	Nicotinamide adenine dinucleotide
NADPH	Nicotinamide adenine dinucleotide phosphate
NF-kB	nuclear factor kappa-light-chain-enhancer
NLPR3	NLR family pyrin domain containing 3
NMR	nuclear magnetic resonance spectroscopy
NO	nitric oxide
OVA	Ovoalbumin
OXPHOS	oxidative phosphorylation
PCS	p-Cresol sulfate
PD-1	Program cell death 1
PD1L	Program cell death 1 ligand
pDCs	plasmacytoid DCs
PEP	phosphoenolpyruvate
PFK	phosphofructose kinase
PGD^2^	Prostaglandin D2
PGE^2^	prostaglandin E2
Phe	phenylalanine
PK	pyruvate kinase
PKM2	pyruvate kinase muscle isozyme
PPA	peroxisome proliferator-activated receptors
PPP	pentose phosphate pathway
PRR	pattern recognition receptor
PUFAs	polyunsaturated FAs
RORyt	Retinoic acid receptor related orphan receptor gamma
ROS	reactive oxygen species
S1P	sphingosine 1-phosphate
SARS-CoV-2	Systemic acute respiratory syndrome coronavirus 2
SCD	stearoyl-CoA desaturase
SCFAs	short chain fatty acids
SERCA	Sarcoendoplasmic reticulum Ca 2^+^ - ATPase
SFAs	saturated fatty acids
SHMT2	Serine hydroxymethyl transferase
SOD	superoxide dismutase
ST2	Interleukin 1 receptor-like 1
STAT3	Signal transducer and activator of transcription 3
T2D	type 2 diabetes
TAMs	tumor associated macrophages
Tbet	T box transcription factor 21
TCA	tricarboxylic acid cycle
TCR	T cell receptor
Teff	T effector cell
TGF-β	transforming growth factor beta
Th2	type 2 T helper cells
Thr	threonine
TLR	toll-like receptors
TLR-2	toll-like receptor 2
TLR-4, TLSP	toll like receptor 4, Thymic stromal lymphopoietin
TNF-α	tumor necrosis factor alpha
Treg	regulatory T cell
TRM	tissue-resident memory T
Trp	tryptophan
Tyr	tyrosine
VAT	visceral adipose tissue
VEGFA	vascular endothelial growth factor A
VLDL	very low density lipoproteins
